# The GRACE Scale in the Prognosis of Patients with Takotsubo Syndrome

**DOI:** 10.1155/2020/4340930

**Published:** 2020-04-24

**Authors:** Malgorzata Zalewska-Adamiec, Lukasz Kuzma, Slawomir Dobrzycki, Hanna Bachorzewska-Gajewska

**Affiliations:** ^1^Department of Invasive Cardiology, Medical University of Bialystok, Białystok, Poland; ^2^Department of Clinical Medicine, Medical University of Bialystok, Białystok, Poland

## Abstract

**Background:**

The prognosis of Takotsubo syndrome (TTS) is comparable to that of the non-ST-elevation myocardial infarction (NSTEMI). The GRACE scale is used to assess the risk of premature and long-term mortality in patients with NSTEMI in order to select the most favorable treatment strategy.

**Methods:**

101 patients with TTS hospitalized in four centers of invasive cardiology in Podlaskie Voivodeship during the period 2008–2012 were included in the study. The patients were divided into two groups: I—52 patients (GRACE ≤ 140 points) and II—49 patients (GRACE > 140 points).

**Results:**

The mean GRACE score in the study group was 138.66. The in-hospital stay of Takotsubo in the patients with higher GRACE scores was associated with higher incidence of pneumonia (36.7% vs 7.69%, *p*=0.0004), rhythm abnormalities (17.3% vs 3.85%, *p*=0.026), and serious complications (cardiogenic shock, pulmonary edema, and sudden cardiac arrest) (30.6% vs 5.77%, *p*=0.001). The mean observation period was 7.2 years. A significantly higher risk of 6-month (18.37% vs 3.85%, *p*=0.019), 1-year (22.45 vs 3.85%, *p*=0.005), 3-year (40.82 vs 3.85%, *p* < 0.0001), 5-year (42.86% vs 3.85%, *p* < 0.0001), and 7-year mortalities (53.06% vs 9.62%, *p* < 0.0001) was observed in the group of patients with a GRACE score ≥140. At multivariate analysis including low BMI, low eGFR, and a higher GRACE score, all these factors were independent predictor of death (*p*=0.042; *p*=0.010; *p*=0.041). The ROC curve presents the discriminatory scores of the GRACE scale for the follow-up prognostication. The area under ROC curve (AUC) for the GRACE scale was 0.805 (95% CI: 0.718–0.892, *p* < 0.0001), with a cut-off value of 153 points, sensitivity of 74%, and specificity of 77% for TTS.

**Conclusion:**

The GRACE scale is highly valuable for the prognostication of death risk in patients with TTS in the early and long-term observation.

## 1. Introduction

Takotsubo syndrome (TTS) consists in the disorders related to the intermittent contractility of left ventricular wall induced by stress, with a significant increase of cardiac enzymes, ischemic changes in electrocardiogram (ECG) records, and lack of significant coronary artery stenosis. TTS is relatively rare and diagnosed in approximately 1-2% of the patients hospitalized for myocardial infarction [1, 2].

For years, TTS was considered a benign disease; however, numerous case reports and registry studies have reported the possibility of highly severe complications, such as severe heart failure, rhythm abnormalities including sudden cardiac arrest and cardiac rupture, in the acute phase of the disease. Takotsubo may relapse in the follow-up period, and the prognosis is comparable to that of the non-ST-elevation myocardial infarction (NSTEMI) [3–6].

Scales to assess prognosis and to facilitate the qualification of patient for various therapeutic methods have been developed for many disease entities [7–9]. In the case of Takotsubo, so far only the TTS diagnosis scale, InterTAK (InterTAK Diagnostic Score) [10], has been developed, while there is no scale dedicated to the assessment of the prognosis of Takotsubo patients. The GRACE scale (Global Registry of Acute Coronary Events) has been used for years in patients with NSTEMI in order to select the most favorable treatment strategy and to assess the risk of death in long-term follow-up [11, 12]. As the clinical presentation of TTS is similar to that of acute coronary syndromes and the long-term prognosis is comparable to NSTEMI, a possibility exists that the GRACE may be helpful in the assessment of Takotsubo prognosis.

The study objective was to verify if the GRACE scale can be applied for the assessment of early and long-term deaths in Takotsubo patients.

## 2. Methods

### 2.1. Study Population

A total of 101 patients with TTS were hospitalized in four centers for invasive cardiology in Podlaskie Voivodeship during the period 2008–2012. TTS was diagnosed based on the applicable Mayo Clinic criteria [13], including 6 patients who were diagnosed with TTS despite the presence of significant changes in the coronary arteries. The new Takotsubo criteria contained in the position of the European Society of Cardiology [14]; it is possible to diagnose TTS with the presence of significant coronary stenosis; therefore, all 101 patients were included in the study.

The GRACE scores were calculated for all patients taking into account the age, heart rate, systolic blood pressure, plasma creatinine concentration, Killip–Kimball class at admission, cardiac arrest at admission, ST-segment changes, and elevated myocardial necrosis markers. Scores on the GRACE 2.0 scale and the risk of in-hospital, 6-month, 1-year, and 3-year death were estimated using the calculator available on the http://www.gracescore.org website.

The patients were divided into 2 groups depending on the score on the GRACE scale as follows: Group I—52 patients (GRACE score ≤ 140 points) and Group II—49 patients (GRACE score > 140 points).

### 2.2. Follow-Up Examination

A long-term observational study after 7 years was conducted based on data concerning deaths obtained from the Division of Data Sharing of the Ministry of Digitalization (Department of National Systems). Mortality due to any cause was assumed as the main endpoint of the study.

### 2.3. Statistical Analysis

The obtained data were subjected to statistical analysis. The quantitative data were compared by Student's test and the Mann–Whitney *U* test, whereas qualitative data were compared by the ch2 test and Fisher's test. The sensitivity and specificity of the Grace scale were assessed using the ROC curve (Receiver Operating Characteristic curve). The survival analysis was conducted with the Kaplan–Meier method, and the comparison of groups with the log-rank test. The multivariate analysis was conducted with the logistic regression method. Aside from the factors in which the tested groups differed significantly, the analysis also included the factors of potential significance in the prognosis of patients with acute coronary syndromes. The value of <0.05 was assumed as statistically significant. The statistical analysis was performed using the STATISTICA 13.1 software.

The study was carried out upon approval from the Bioethics Committee of the Medical University of Bialystok.

## 3. Results

### 3.1. Studied Population and Group Comparison

Table 1 presents the characteristics of the entire Takotsubo group (101 patients) and the study groups with lower and higher GRACE scores. The study group comprised 87% of women, and the mean age of patients was 68. The mean GRACE score was 138.66 (SD—36.75), while the median was –138.

Patients with a GRACE score >140 were older and had a lower body mass index (BMI). They had a higher incidence of arterial hypertension, diabetes, chronic obstructive pulmonary disease (COPD), cancer, and chronic kidney disease, in addition to lipid disorders and a burdened family history.

Patients with a higher GRACE had significantly lower left ventricular ejection fraction (LVEF). Furthermore, in these patients, cardiac catheterization more frequently demonstrated atherosclerotic changes in the coronary arteries (irrelevant and significant), while the ECG records more frequently demonstrated ST-segment elevation.

In the group of patients with a GRACE score >140, the results of the laboratory tests demonstrated higher values of inflammatory parameters and higher levels of cardiac enzymes, glucose on admission, and creatinine, whereas the concentrations of all lipid fractions were lower.

In the group with a GRACE score ≥140, significantly lower values of the systolic arterial pressure and higher heart rates were observed on admission. The in-hospital stay of the Takotsubo patients with higher GRACE scores was associated with higher incidence of pneumonia, rhythm abnormalities, cardiogenic shock, pulmonary edema, and sudden cardiac arrest. Cardiac rupture was noted only in this group, in 3 patients.

### 3.2. GRACE Scale and Mortality

**Figure 1:**
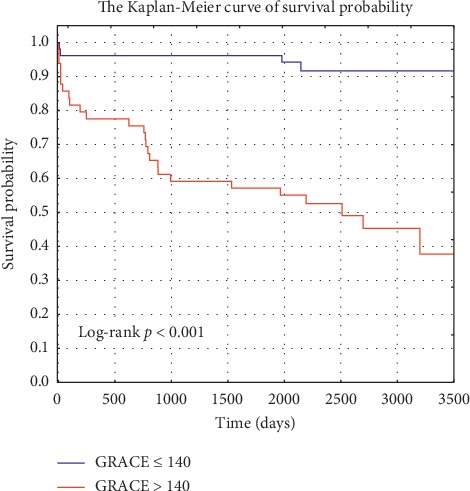
The Kaplan–Meier curve of survival probability of patients with Takotsubo (*n* = 101).

In the long-term study, the mean observation period was 87 months (7.2 years). The risk of death in 1-year and 3-year follow-up periods estimated using the GRACE calculator was higher in the group of patients with higher GRACE scores. In the observational study, a significantly higher risk of 6-month and 1-, 3-, 5-, and 7-year mortality was observed in the group of patients with a GRACE score ≥ 140. Data on mortality and the Kaplan–Meier curves are presented in the table (Table 2) and in the figure (Figure 1).

In the multivariate analysis performed by the logistic regression method, the risk factors for death in long-term follow-up are low BMI (<20 kg/m^2^), low GFR (<60 m/min/1.72 m^2^), and more points on the GRACE scale (Table 3).

### 3.3. Predictive Value of the GRACE Scale

**Figure 2:**
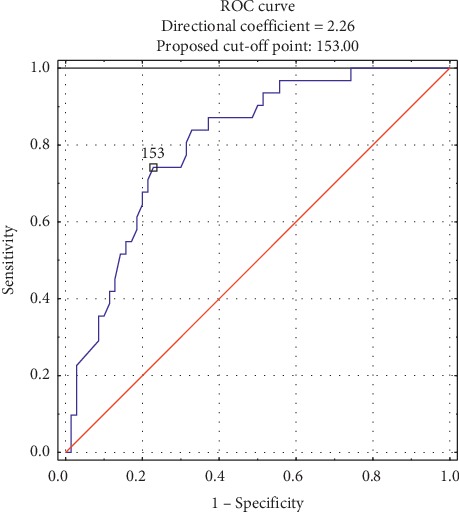
Receiver operating characteristic (ROC) curves for GRACE risk score in the prediction of 7-year mortality in patients with Takotsubo syndrome 1 − Specificity.

In Figure 2, the ROC curve presents the discriminatory scores of the GRACE scale in the predicting long-term prognosis. The area under ROC curve (AUC) for the GRACE scale was 0.805 (95% confidence interval [CI] 0.718–0.892, *p* < 0.001), with a cut-off value of 153 points, and the sensitivity was 74%, and the specificity was 77% for TTS (Figure 2).

## 4. Discussion

The first case report on the “apical ballooning cardiomyopathy” was published by Sato et al. [15] almost 30 years ago. Over the years, large international Takotsubo registers have been created, on the basis of which many publications have been developed. Initially, Takotsubo was considered to be a benign clinical entity with good prognosis. Left ventricular wall motion abnormalities generally recover within few weeks, but it was observed that serious life-threatening complications in the form of acute respiratory failure, cardiogenic shock, serious arhytmia, and cardiac rupture may occur in the acute phase of the disease [1, 2]. There is a greater risk of complications in takotsubo in patients with impaired renal function [16–18]. The pathomechanism of the disease has not yet been fully explained, and the methods to effectively treat those patients to prevent complications in the acute phase and the recurrence of the disease in the follow-up period remain unknown.

Based on the large European Takotsubo registry, the InterTAK score [10] has been developed for the diagnosis of TTS; however, no scale that would assist in the therapeutic decision-making and in assessing the prognosis of Takotsubo patients has been developed yet. Considering that the clinical presentation of Takotsubo bears resemblance to acute coronary events, the present study assessed the use of the GRACE scale in the prognosis of TTS patients.

The GRACE scale was developed on the basis of a large international registry of acute coronary syndrome, GRACE (The Global Registry of Acute Coronary Events). Over 102,000 patients with ACS from 250 hospitals in 30 countries were included in this study. The hospital and long-term therapeutic results of patients with acute coronary events were observed, and using these data, the point risk scale was developed [11]. The current GRACE 2.0 scale version is available as an online calculator (GRACE 2.0 ACS Risk Calculator). The European Society of Cardiology has been recommending the use of the GRACE scale for risk stratification and the selection of appropriate treatment strategy for patients with NSTEMI since 2010 [19, 20].

Santoro et al. demonstrated the value of persistent ST-segment elevation in electrocardiography as a predictor of complications during hospitalization and adverse events in an observational study [21]. Takotsubo patients included in the Italian-German GEIST registry were classified as low-risk and high-risk patients based on 4 variables that were identified as independent predictors of hospital complications and adverse events in follow-up (male gender, history of neurological disorders, right ventricular involvement, and left ventricular ejection fraction (LVEF) [22].

In a group of 537 Takotsubo patients, Scudiero et al. [23] noted a significantly higher mortality in the group of patients with a GRACE score > 140 (17% vs 8%) after an average follow-up period of 40 months. In the presented analysis, the differences in the deaths rates of patients with a GRACE score > 140 and ≤140 in a similar 3-year follow-up period were even higher, with the mortality rate amounting to 40.8% vs 3.85%. In addition, in a group of 561 TTS patients, Scudiero et al. [24] determined a significant discriminatory value of the GRACE score for the prognostication of mortality and cerebrovascular incidents after an average follow-up period of 29 months. For all-cause mortality, the area under ROC curve (AUC) was calculated as 0.67 (95% CI 0.60–0.73; <0.001). In this paper, the discriminatory value of the GRACE scale was even stronger (AUC—0.805, 95% CI 0.718–0.892, <0.001), which is the result of the high mortality of our patients with a higher GRACE score and a longer observation period.

In addition, the applicability of the CHA2DS2-VASc scale, which is used to assess the thromboembolic risk in patients with atrial fibrillation, was evaluated for TTS. In a group of 371 patients, Parodi et al. [25] determined the significant discriminatory ability of the CHA2DS2-VASc scale for the prediction of mortality rate and cardiac and cerebrovascular events. At the same time, a comparison of both scales in the Takotsubo group demonstrated the advantage of the GRACE scale in predicting the prognosis of TTS patients.

It is beyond any doubt that a scale is needed for the assessment of and treatment qualification in the case of Takotsubo syndrome, as well as other acute cardiologic entities. However, until a new specific scale dedicated to TTS will be developed and validated, the GRACE score could be useful for the TTS management. Further research assessing the usefulness of the GRACE scale in the assessment of prognosis of Takotsubo patients is needed to more precise estimation of death risk in these patients in long-term follow-up. Adaptation of the scale to TTS may facilitate the treatment decision-making for Takotsubo patients and in particular the decision on the minimum time of hospitalization, including observation in the intensive cardiology care unit.

## 5. Conclusions

The GRACE scale is highly valuable for the prognostication of death risk in patients with TTS in the early and long-term follow-up period. Further analyses of the application of GRACE scale and other scales used for the assessment of potential risks should be performed on more extensive groups of Takotsubo patients.

### 5.1. Study Limitation

The greatest study limitation is the inclusion of a low number of TTS patients in the study. Another limitation is that the analysis is retrospective in nature. In the observational study carried out on the PESEL database, only the information on the death dates of the patients was obtained, but the death causes of the deaths were unknown.

## Figures and Tables

**Table 1:** Results—comparison of 2 groups of patients with Takotsubo (*N* = 101).

GRACE	TTS *N* = 101 (SD) *N* (%)	TTS ≤ 140 *N* = 52 (SD) *N* (%)	TTS > 140 *N* = 49 (SD) *N* (%)	*p*
*Clinical and demographic characteristics*:				
Age (years)^GRACE^	68.01 (13.88)	58.62 (12.62)	77.98 (6.26)	<0.0001
Female sex (%)	88	45	43	0.855
	87.13%	86.54%	87.76%	
Body mass index (BMI) kg/m^2^	25.79 (4.83)	26.88 (4.54)	24.69 (4.97)	0.038
History of malignancy (%)	5	0	5	0.018
	4.95%	0.0%	10.20%	
History of hypertension (%)	65	28	37	0.023
	64.36%	53.85%	75.51%	
Hyperlipidemia (%)	36	25	11	0.007
	35.64%	48.08%	22.45%	
Smoking (%)	22	13	9	0.419
	21.78%	25.0%	18.37%	
Family history of coronary artery disease (%)	16	13	3	0.009
	15.84%	25.0%	6.12%	
Diabetes mellitus (%)	12	3	9	0.05
	11.88%	5.77%	18.37%	
Anxiety/depression (%)	8	4	4	0.930
	7.92%	7.69%	8.16%	
Thyroid disorders (%)	24	12	12	0.868
	23.76%	23.08%	24.49%	
Chronic kidney disease (%)^GRACE^	12	0	12	0.0001
	11.88%	0.0%	24.49%	
COPD (%)	11	3	8	0.088
	10.89%	5.77%	16.33%	

*Diagnostic tests (echocardiography*, *coronarography*, *ECG)*:				
Left ventricular ejection fraction on admission (%)	39.63 (9.72)	43.12 (9.92)	36.83 (9.09)	0.0015
No atherosclerotic changes in coronary arteries (%)	42	28	14	0.009
	41.58%	53.85%	28.57%	
Insignificant stenoses (%)	53	23	30	0.087
	52.47%	44.23%	61.22%	
Significant stenoses (%)	6	1	5	0.078
	5.94%	1.92%	10.20%	
ECG—ST-segment elevation (%)^GRACE^	73	31	42	0.003
	72.28%	59.62%	85.71%	
QTc on admission (ms)	468.09 (37.70)	461.76(36.57)	479.56(39.37)	0.135
QTc after a few days (ms)	478.90 (82.06)	460.37(99.16)	503.61(46.94)	0.095

*Laboratory parameters*:				
Hemoglobin (mg/dl)	13.29 (1.65)	13.42 (1.53)	13.15 (1.79)	0.419
Erytrocytes(x106/*μ*l)	4.47 (0.65)	4.52 (0.57)	4.41 (0.72)	0.382
Hematocrit (%)	39.37 (6.08)	39.31 (6.64)	39.31 (5.57)	0.924
Leukocytes (x103/*μ*l)	9.89 (4.03)	8.36 (2.27)	11.52 (4.85)	<0.0001
Glucose on admission (mg/dl)	126.06 (46.06)	107.87(20.14)	145.01(57.27)	<0.0001
Creatinine (mg/dl)^GRACE^	0.93 (0.46)	0.79 (0.25)	1.08 (0.59)	0.0013
eGFR MDRD (m/min/1.72 m^2^)	73.72 (27.58)	84.79 (25.44)	61.96 (25.29)	<0.0001
CK (IU/l)	428 (908.2)	279.98(311.2)	553.60(1202.51)	0.208
Troponin (significant increase) (%)^GRACE^	88	46	42	0.680
	87.13%	88.46%	85.71%	
Troponin—mean concentration (ng/ml)	5.72 (11.60)	4.84 (6. 25)	6.64 (15.42)	0.447
Total cholesterol (mg/dl)	181.2 (42.07)	192.07(46.13)	168.76(33.89)	0.009
LDL (mg/dl)	110.3 (38.72)	121.35(42.23)	96.65 (29.68)	0.003
HDL (mg/dl)	51.65 (17.80)	52.31(16.97)	50.83 (19.19)	0.709
Triglycerides (mg/dl)	98.28 (52.25)	100.83(54.14)	95.28 (51.14)	0.719
CRP (mg/l)	40.95 (64.92)	16.61 (19.73)	59.21 (80.48)	0.006

*Clinical in-hospital stay and mortality*:				
RRs on admission^GRACE^	128.8 (27.08)	135.31(25.58)	121.94(27.46)	0.012
HR on admission^GRACE^	84.39 (19.04)	77.54 (16.41)	91.65 (19.28)	0.0001
Retrosternal chest pain (%)	82	44	38	0.363
	81.19%	84.62%	77.55%	
Dyspnea (%)	13	4	9	0.109
	12.87%	7.69%	18.37%	
Cardiac arrest, pulmonary oedema, cardiogenic shock (%)^GRACE^	18	3	15	0.001
	17.82%	5.77%	30.61%	
Cardiac rupture (%)	3	0	3	0.070
	2.97%	0.0%	6.12%	
Pneumonia (%)	21	4	18	0.0004
	20.79%	7.69%	36.73%	
Rhythm disturbances (%)	11	2	9	0.026
	10.89%	3.85%	17.31%	

*Pharmacological treatment at discharge*				
Aspirin	96	51	45	0.148
	95.05%	98.08%	91.84%	
ACEI/ARB	87	47	40	0.203
	86.14%	90.38%	81.63%	
*β*-blockers	84	47	37	0.046
	83.17%	90.38%	75.51%	
Statin	86	48	38	0.037
	85.15%	92.3%	77.55%	

COPD—chronic obstructive pulmonary disease, ECG—electrocardiogram, eGFR—estimated glomerular filtration rate, CK—creatine kinase, LDL—low-density lipoprotein, HDL—high-density lipoprotein, CRP—c-reactive protein, RR—blood pressure, HR—heart rate, ACEI—angiotensyn converting enzyme inhibitor, and ARB—angiotensin II receptor blocker. ^*∗*^Factors constituting the GRACE scale.

**Table 2:** Results—comparison of estimated and observed mortality of 2 Takotsubo groups (*n* = 101).

	TTS GRACE points (medium)—138.7 *N* = 101		TTS ≤ 140 (medium—108.9) *N* = 52		TTS > 140 (medium—170.2) *N* = 49		*p*
*GRACE—estimated risk of death (medium)*							
In-hospital (%)	11.96		3.15		21.31		0.004
6 months (%)	19.58		6.04		33.96		0.0004
1 year (%)	22.31		7.27		38.27		0.0001
3 year (%)	38.74		15.91		62.96		<0.0001

*GRACE—observed mortality*							
	*n*	%	*n*	%	*n*	%	
In-hospital	6	5.94	2	3.85	4	8.16	0.359
1 month	8	7.92	2	3.85	6	12.24	0.118
3 months	9	8.91	2	3.85	7	14.29	0.065
6 months	11	10.89	2	3.85	9	18.37	0.019
1 year	13	12.87	2	3.85	11	22.45	0.005
3 year	22	21.78	2	3.85	20	40.82	<0.0001
5 year	23	22.77	2	3.85	21	42.86	<0.0001
7 year	31	30.69	5	9.62	26	53.06	<0.0001

**Table 3:** Univariable and multivariable regression analysis of risk factors of death in Takotsubo (*n* = 101).

Predictor	Univariable			Multivariable		
	Odd ratio	95% Cl^*∗*^	*p*	Odds ratio	95% Cl^*∗*^	*p*
Age	0.942	0.904–0.982	**0.005**			
BMI < 20	1.152	1.025–1.294	**0.017**	**0.857**	**0.738–0.995**	**0.042**
eGFR MDRD	1.035	1.014–1.056	**<0.001**	**0.954**	**0.920–0.989**	**0.010**
Hypertension	0.652	0.262–1.623	0.357			
Smoking	1.389	0.346–5.576	0.643			
EF < 40%	1.059	1.010–1.110	**0.018**			
Hemoglobin	1.086	0.836–1.411	0.538			
CRP	0.993	0.986–1.001	0.104			
Troponin	0.975	0.937–1.014	0.208			
Creatine kinase	0.999	0.998–1.000	0.051			
LDL	1.024	1.006–1.041	**0.008**			
GRACE points	0.962	0.946–0.979	**<0.001**	**1.035**	**1.001–1.069**	**0.041**

Cl–confidence interval.

## Data Availability

The clinical data used to support the findings of this study are included within the article. The particular data are available from the corresponding author upon request.
